# Amalgam Electrode-Based Electrochemical Detector for On-Site Direct Determination of Cadmium(II) and Lead(II) from Soils

**DOI:** 10.3390/s17081835

**Published:** 2017-08-09

**Authors:** Lukas Nejdl, Jindrich Kynicky, Martin Brtnicky, Marketa Vaculovicova, Vojtech Adam

**Affiliations:** 1 Central European Institute of Technology, Brno University of Technology, Purkynova 123, 612 00 Brno, Czech Republic; lukasnejdl@gmail.com (L.N.); martin.brtnicky@mendelu.cz (M.B.); marketa.vaculovicova@mendelu.cz (M.V.); 2 Department of Chemistry and Biochemistry, Mendel University in Brno, Zemedelska 1, 613 00 Brno, Czech Republic; 3 Department of Microelectronics, Faculty of Electrical Engineering and Communication, Brno University of Technology, Technicka 10, 616 00 Brno, Czech Republic; 4 Department of Geology and Paedology, Faculty of Forestry and Wood Technology, Mendel University in Brno, Zemedelska 1, 613 00 Brno, Czech Republic

**Keywords:** amalgam electrodes, electrochemistry, heavy metals, soil, turbid sample

## Abstract

Toxic metal contamination of the environment is a global issue. In this paper, we present a low-cost and rapid production of amalgam electrodes used for determination of Cd(II) and Pb(II) in environmental samples (soils and wastewaters) by on-site analysis using difference pulse voltammetry. Changes in the electrochemical signals were recorded with a miniaturized potentiostat (width: 80 mm, depth: 54 mm, height: 23 mm) and a portable computer. The limit of detection (LOD) was calculated for the geometric surface of the working electrode 15 mm^2^ that can be varied as required for analysis. The LODs were 80 ng·mL^−1^ for Cd(II) and 50 ng·mL^−1^ for Pb(II), relative standard deviation, RSD ≤ 8% (*n* = 3). The area of interest (Dolni Rozinka, Czech Republic) was selected because there is a deposit of uranium ore and extreme anthropogenic activity. Environmental samples were taken directly on-site and immediately analysed. Duration of a single analysis was approximately two minutes. The average concentrations of Cd(II) and Pb(II) in this area were below the global average. The obtained values were verified (correlated) by standard electrochemical methods based on hanging drop electrodes and were in good agreement. The advantages of this method are its cost and time effectivity (approximately two minutes per one sample) with direct analysis of turbid samples (soil leach) in a 2 M HNO_3_ environment. This type of sample cannot be analyzed using the classical analytical methods without pretreatment.

## 1. Introduction

Toxic metals pollution, as a global problem, includes contamination of air, soil, and surface waters as a consequence of traffic, heavy industry, and mining. Metal elements such as lead and/or cadmium, or their compounds are extremely toxic for the environment even in trace concentrations [1,2]. The issue of metal contamination does not affect the source alone. Depending on several factors such as particle size distribution, organic matter content, and/or vegetation [3], these metals can move long distances and contaminate the land even thousands of kilometres from the source. For this reason, it is necessary to find easy-to-use and inexpensive analytical tools for detection of metals [4].

Presently, various traditional analytical methods for trace cadmium and lead in soils or environmental samples are often employed, such as flame atomic absorption spectrometry (F-AAS), electrothermal atomic absorption spectrometry (ET-AAS), and inductively coupled plasma atomic emission spectroscopy (ICP-MS). Voltammetric/polarographic approaches are also suitable for these purposes. The basic setup for most of the analyses require the sample introduction as a liquid solution and thus, for solid matrices, an acid digestion (extraction) procedure becomes mandatory. Sample digestion is mainly carried out by a fusion or a wet procedure based on an acid digestion with a heated mixture of mineral acids. The sample is mineralized by microwave (thermal) reactor equipped with temperature and pressure control assisted by common acids (nitric, perchloric, sulphuric, and hydrochloric acid) [5]. For voltammetric analyses it is recommended to treat the wastewater after sampling by nitric acid (pH 2). The acidic sample is filtered through a 0.45 µm membrane filter and subsequently irradiated by UV for digestion [6]. This process destroys the organic compounds-metal complexes and the metals pass into their ionic form. In the case of low efficiency of UV-digestion, concentrated nitric acid together with concentrated perchloric acid are added to the sample. The sample is evaporated and then diluted with the desired electrolyte. Solid samples (soil and waste sludge) are first dried and then treated in a similar way as in the previous case.

Voltammetry or polarography is the most commonly used electrochemical method usually used for determination of substances dissolved in aqueous solutions or in organic solvents [7,8]. Hanging mercury drop electrodes (HMDEs) are commonly used for these applications [9,10,11,12]. The disadvantages are the small extent of the anodic region (limitation of mercury oxidation), toxicity, impossibility to be placed into the flow-through arrangement, susceptibility of hanging drop to upheaval and impurities. For those reasons, this electrode cannot be used for on-site analyses, unlike solid ones. State-of-art solid electrodes are mostly composed of carbon (glassy carbon, boron-doped diamond, carbon nanotubes, carbon nanosheets, vapour deposited carbon films, and various composite [13]), or combined with a variety of nanoparticles [14,15] and nanostructured materials [16,17]. For example, Cd(II) and Pb(II) can be detected by glassy carbon electrode modified with bismuth nanoparticles [18,19] or carbon nanotubes paste electrode modified with Amberlite IR-120 [20].

Solid electrodes, which can be used both in the laboratory and for on-site conditions are continually being developed. Suitable solid electrodes for the more positive potential region are based on solid materials such as glassy carbon [21], carbon paste [22], noble metals [23], composites [24], conducting polymers [25], and various combinations with nanomaterials [26,27]. For environmental applications, a miniaturized electrochemical detection with solid electrode systems is the most suitable because of the ability to analyze turbid samples.

Screen-printing microfabrication technology is nowadays well established for the production of thick-film electrochemical transducers usually called screen-printed electrodes (SPE). A small dosage area enables very low sample consumption, in some cases sample volumes can be smaller than 10 μL. The compact size and excellent selectivity and sensitivity enable incorporation of SPE into the flow automatically [28] or robotic systems [29] for on-site analysis. Furthermore, these electrodes are suitable for environmental monitoring of metal ions [30] and multiplexing (sensor array) [31]. The disadvantage of SPE is mainly its single use of each electrode and a large cost of screen-printing microfabrication equipment. Further, organic solvents and very low pH can be responsible for the dissolution of insulating inks or surface of the electrode and consequently limiting its performance.

Electrodes, where the solid surface is covered by mercury (Ag/Hg, Pt/Hg, Au/Hg, Ir/Hg, and Cu/Hg), represent some kind of intermediate between the solid surface and pure mercury [32]. The amalgam electrode is an alternative to HMDE, because this electrode not only shows similar sensitivity and properties as HMDE, but also negligible toxicity, easy manipulation as it can be used multiple times, and can be miniaturized [33,34]. It is impossible to immerse the HMDE into a solution containing solid impurities (due to its mechanical sensitivity) and therefore, conventional electrochemical measurement by polarography is prevented. This problem can be resolved by using an amalgam electrode. Nowadays, there are many types of amalgam electrodes (liquid, paste, and solid) as shown in [35]. The main advantage of a copper amalgam electrode is its price and availability. In some cases, the Cu/Hg electrodes may have better electrode properties compared to Ag/Hg and Au/Hg electrodes. For example, Jelen et al. used the copper solid amalgam electrode for detection of DNA at subnanomolar concentrations in the presence of copper [36]. Further, a similar type of electrode was used for detection of fungicide [37], herbicide [38], metallothionein [4], and phytochelatins [39]. The amalgam electrodes (Ag/Hg, Pt/Hg, Au/Hg, Ir/Hg, and Cu/Hg) differ from each other by the range of working potentials as shown here [4].

Solid electrodes and SPE have been developed for a variety of applications in environmental, industrial, biological, and clinical areas. However, until now, there are no studies addressing direct electrochemical detection of turbid samples in field conditions (on-site). It is known that the acid used in the extraction (metals from soil), together with the colloidal particles, and various organic substances contained in the soil sample prevents direct detection using HDME and most of the solid electrodes. For example, when the pH value is low (3.5), the H^+^ is reduced on the surface of working electrode to H_2_. The hydrogen evolution reaction hinders the deposition of the target metal ion on the electrode surface [40,41]. For these purposes, it may require the use of an amalgam electrode as we demonstrated in this work.

Here, a laboratory-made amalgam working electrode (Cu/Hg-WE), together with a portable computer and potentiostat, was used for the sensitive analysis of Cd(II) and Pb(II) on-site in Dolni Rozinka (Czech Republic). These metals were extracted by 2 M HNO_3_ and analysed in 0.1M acetate buffer with 5% HCl (*v*/*v*) by differential pulse voltammetry.

## 2. Materials and Methods

### 2.1. Chemicals and Material

All chemicals including lead(II) nitrate, cadmium(II) sulphate, mercury(II) nitrate, atomic absorption spectrometric standard of metals (Ni(II), Zn(II), Cu(II), As(III), Hg(I), Se(II), and Fe(II)), and nitric acid (HNO_3_, ultraclean) were purchased from Sigma-Aldrich (Sigma-Aldrich, St. Louis, MO, USA) unless noted otherwise. Stock solutions were prepared in ACS-purity water immediately prior to use.

### 2.2. Modification of Electrolytic Copper Wire to Serve as Working Electrode (Cu/Hg-WE)

Copper wires (Thermo scientific, Cambridge, UK) were used as working electrodes after modification. Briefly, the copper wire was immersed into 0.01 M Hg(NO_3_)_2_ solution, prepared by the dissolution of 86 mg mercury(II) nitrate in 25.0 mL of acidified (5% HNO_3_, *v*/*v*) Milli-Q water. The electrodes were immersed in this solution for 180 s, which resulted in the formation of a thin-film of amalgam on the surface.

### 2.3. Electrochemical Determination of Cadmium and Lead by the Amalgam Electrode

Electrochemical detection was performed using a three-electrode system. A solid Cu/Hg-WE electrode with dimensions of 0.3 mm (diameter) × 15 mm (length) was used. An Ag/AgCl/3 M KCl electrode was the reference (RE) and a platinum electrode was auxiliary (CE). Then, 200 mg of soil samples or 1 mL of wastewater were added into the electrochemical cell with 0.1 M acetate buffer containing 1 M HNO_3_ and 5% HCl (*v*/*v*), with a 2200 µL total volume. Plastic UV/VIS semi-micro cuvette with dimensions of 12.5 × 12.5 × 45.0 mm (Brand, Wertheim, Germany) was used as an electrochemical cell. The electrode holder was designed and 3D-printed by a Profi3DMarker printing system (3Dfactories, Straznice, Czech Republic). Electrochemical determination was carried out by differential pulse voltammetry (DPV). Changes in the electrochemical signals were recorded with potentiostat (width: 80 mm; depth: 54 mm; height: 23 mm) 910 PSTAT mini (Metrohm, Herisau, Switzerland) and the results were evaluated by PSTAT software 1.1 Build 120217 (Metrohm, Herisau, Switzerland) using a portable computer Acer Iconia (Acer, Taipei, Taiwan). The parameters of the DPV measurement were as follows: initial potential −1.1 V; end potential −0.2 V; step potential 5 mV; modulation amplitude 10 mV; modulation time 10 ms, deposition time 60 s, deposition potential −1.1 V, and interval time 0.2 s.

### 2.4. Electrochemical Determination of Cadmium and Lead by HMDE

Determination of cadmium and lead by differential pulse voltammetry (standard method) was performed by 797 VA Computrace instrument connected to 813 Compact Autosampler (Metrohm, Herisau, Switzerland), using a standard cell with three electrodes. The three-electrode system consisted of HMDE with a drop area of 0.4 mm^2^ as the working electrode, an Ag/AgCl/3 M KCl reference electrode and platinum as the auxiliary electrode. 797 VA Computrace software by Metrohm CH was employed for data processing. The analysed samples were deoxygenated prior to measurements by purging with argon (99.999%). Acetate buffer (0.2 M CH_3_COONa and 0.2 M CH_3_COOH, pH 5) was used as a supporting electrolyte. The supporting electrolyte was replaced after each analysis. The parameters of the measurement were as follows: purging time 90 s, deposition potential −1.1 V, accumulation time 240 s, equilibration time 5 s, modulation time 0.057 s, interval time 0.04 s, initial potential of −1.3 V, end potential 0.2 V, step potential 0.005 V, modulation amplitude 0.025 V, volume of injected sample: 15 µL, volume of measurement cell 2 mL (15 μL of sample and 1985 μL acetate buffer).

### 2.5. Atomic Absorption Spectrometry (AAS)

Cadmium and lead were determined on an Agilent Technologies 80Z atomic absorption spectrometer (Agilent, Santa Clara, CA, USA) with electrothermal atomization. The spectrometer was operated at the 228.8 nm (Cd^2+^) and 283.3 nm (Pb^2+^) resonance line with a spectral bandwidth of 0.5 nm. The sample volume (20 μL) was injected into the graphite tube. The flow of argon (inert gas) was 300 mL·min^−1^. Zeeman background correction was used with field strength of 0.8 Tesla. The absorption signal was evaluated in peak height mode with seven point smoothing.

### 2.6. Descriptive Statistics

Mathematical analysis of data and their graphical interpretation were realized by Microsoft Excel^®^. Deviations are shown as (standard deviation = SD or relative standard deviation = RSD). The detection limits (LODs) were calculated according to the equation of the calibration curve as (3 SD + shift)/slope. LOD was calculated for the geometric surface of the working electrode 15 mm^2^.

### 2.7. Description of the Studied Sites (Dolni Rozinka, Czech Republic) and Motivation for On-site Analysis

Area of interest (Dolni Rozinka) is located in the Ceskomoravska vrchovina (Bohemian-Moravian Highlands) in the south-eastern part of the district Zdar nad Sazavou, approximately 50 km northwest from Brno and 12 km northwest from Tisnov. Uranium mining in this area has brought substantial negative impacts on the environment such as a high burden of heavy haulage of hazardous materials, an increase in noise, dust, radon release, damage of the landscape, and groundwater contamination. Emission of metals that tend to accumulate in the water, soil, or organisms have to be also mentioned. Adsorption of the contaminants on the surface of the atmospheric aerosol (solid and liquid particles with diameter of less than 2.5 μm), forms stable aerodispersion system, which is characterized by a long dwell time in an atmosphere. In these forms, metals can be moved with air for long distances and contaminate the land even thousands of kilometres from the source. This type of pollution is a global issue and therefore, we have decided to optimize the user-friendly electrochemical method, which can sensitively monitor toxic metals at nanomolar concentrations on-site. This type of electrochemical analysis is primarily designed for aqueous solutions, but thanks to the easy extraction of metals by acids, this method can also be used for a variety of environmental samples, as is evidenced further.

## 3. Results and Discussion

### 3.1. Preparation of Homemade Working Electrode

**Figure 1:**
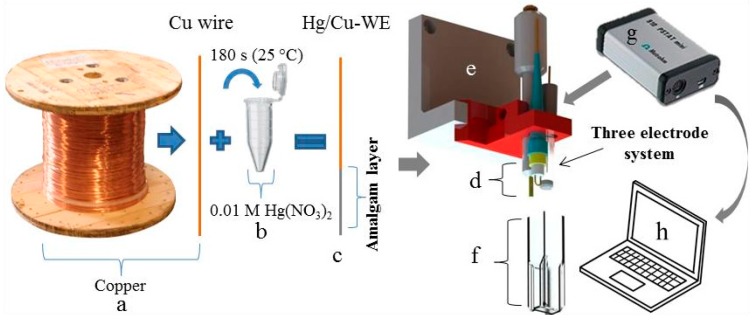
Schematic representation of the preparation of the working amalgam electrode and analytical instrument, (**a**) copper wire and (**b**) 0.01 M solution of Hg(NO_3_)_2_ in which the copper wire was immersed for 180 s and was tempered to 25 °C; (**c**) Scheme of the formed amalgam layer; (**d**) three electrode system (Cu/Hg-WE, Ag/AgCl/3 M KCl-RE and Pt-CE); (**e**) electrode holder printed by 3D printer; (**f**) plastic cuvette serving as an electrochemical cell; (**g**) portable potentiostat; and (**h**) portable computer with PSTAT software.

Electrolytic copper wire (Figure 1a) was immersed into 0.01 M Hg(NO_3_)_2_ for 180 s (Figure 1b). Immediately upon immersion of the copper wire into the solution, the formation of an amalgam layer of silver colour starts on its surface (Figure 1c). Thanks to amalgamation, this type of working electrode (Cu/Hg-WE) was expected to be sensitive to the presence of metal ions [33]. After amalgamation, the electrode (Cu/Hg-WE) was rinsed with distilled water and dried. Together with a reference silver chloride (Ag/AgCl/3 M KCl-RE) and an auxiliary platinum electrode (Pt-CE) a standard three electrode system (Figure 1d) was formed and anchored in a 3D-printed holder (Figure 1e). UV/VIS cuvette with an optical path of 1.0 cm served as the electrochemical cell (Figure 1f). The electrochemical signals were recorded by portable potentiostat 910 PSTAT mini (width: 80 mm; depth: 54 mm; height: 23 mm; Figure 1g) and processed using PSTAT software on a portable computer (Figure 1h).

Freshly prepared electrodes cannot be stored for long periods on air. Storage in solution has not been tested, but is theoretically possible. Our experience shows that the best way was to always make the electrode fresh (180 s immerse in (HgNO_3_)_2_). After preparation, the electrode was polished and validated, using a standard sample (several measurements for activation and control of the stability). A great advantage of the described Cu/Hg-WE electrodes is the practically unrestricted recovery of the amalgam layer. When the performance of the electrode is decreasing, it is sufficient to immerse it again in (HgNO_3_)_2_ solution. In this way, the electrode work surface is restored.

### 3.2. The Optimization of Pb(II) and Cd(II) Detection

**Figure 2:**
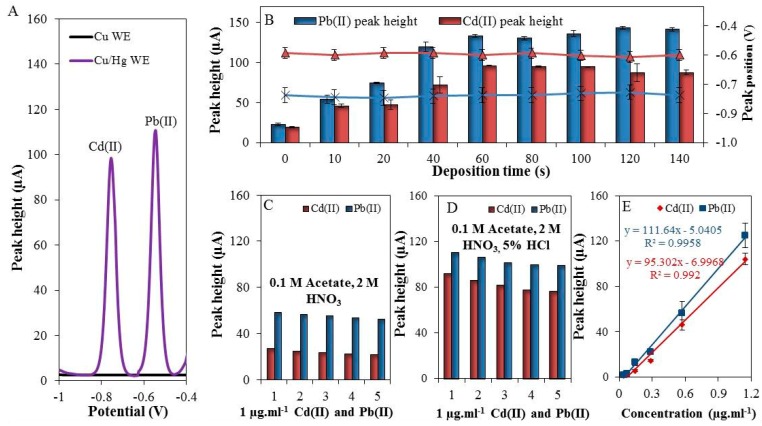
(**A**) Typical differential pulse voltammograms of Cd(II) and Pb(II) measured by modified and unmodified working electrode (WE); (**B**) The dependence of the deposition time (0–140 s) on the electrochemical response of both cadmium (red columns), and lead (blue columns) and on the position of the peaks of Cd(II) (black cross) and Pb(II) (blue triangles); (**C**) Five consecutive measurements of Cd(II) and Pb(II) in 0.1 M acetate with 2 M HNO_3_; (**D**) Five consecutive measurements of Cd(II) and Pb (II) in 0.1 M acetate with 2 M HNO_3_ and 5% HCl; (**E**) The calibration curve of Cd(II) and Pb(II), deposition time 60 s, deposition potential −1.1 V, electrolyte was 0.1 M acetate with 2 M HNO_3_ and 5% HCl, and geometric WE area = 15 mm^2^.

As it was expected, the above-described setup allowed for detection of well-developed Cd(II) and Pb(II) peaks, shown in Figure 2A (black = unmodified and purple = modified WE). The surface of the working electrode was characterized by scanning electron microscope (elemental mapping and X-ray fluorescence) in our previous work [4].

The optimization of experimental conditions of the analysis itself was focused on monitoring of the electrochemical response of the individual elements depending on the increasing deposition time within the range from 0 to 140 s. An increase in the deposition time caused an increase in the electrochemical signal of both Cd(II) and Pb(II) (Figure 2B). The best electrochemical response was achieved at the deposition time of 60 s (Cd(II) concentration of 1 μg·mL^−1^). The signal plateau was reached due to the saturation of the electrode surface. It has to be noted that the benefit of this electrode is in the option to adjust the size of the surface as necessary. The dependence of the peak position on the deposition time was also examined. It was found that the positions of the electrochemical signals for lead (peak position = −0.58 V) and cadmium (peak position = −0.79 V) did not changed, as is shown in Figure 2B (triangle = Pb(II) and cross = Cd(II)).

Furthermore, the repeatability of individual analyses (Cd(II) and Pb(II)) in 0.1 M acetate buffer with 2 M HNO_3_ (extraction agent) was observed. As shown in Figure 2C, the repeatability of five consecutive measurements of Cd(II) and Pb(II) was 3.8 and 7.6% (RSD), respectively. After addition of 5% HCl (*v*/*v*) an increase in electrochemical signal was observed - 3.5-fold in the case of Cd(II) (RSD = 4.3%) and 1.9-fold in the case of Pb(II) (RSD = 6.8%), which is depicted in Figure 2D. In this way, it was demonstrated that Cu/Hg-WE can be used for reliable detection of Cd(II) and Pb(II) in 0.1 M acetate buffer with additions of 2 M HNO_3_ and 5% HCl, which enabled us further experiments with environmental samples.

Next, the electrochemical responses of different concentrations (0 to 2000 ng·mL^−1^) of Cd(II) and Pb(II) were monitored. The optimal conditions were used (deposition time = 60 s, supporting electrolyte = 0.1 M acetate buffer with 2 M HNO_3_ and 5% HCl). Deposition potential was set to −1.1 V [42]. The linear dynamic ranges were 200–1143 ng·mL^−1^ and 200–1143 ng·mL^−1^ for Cd(II) and Pb(II), respectively. The calibration curves were determined (Figure 2E) and the figures of merit are summarized in Table 1. The LODs were 80 ng·mL^−1^ for Cd(II) and 50 ng·mL^−1^ for Pb(II), RSD ≤ 8%, (*n* = 3).

### 3.3. Study of Interfering Substances and Time of Extraction

**Figure 3:**
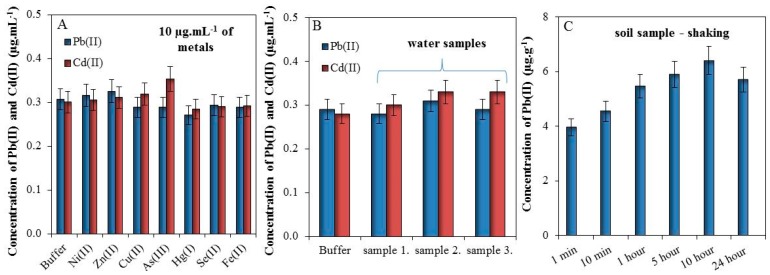
(**A**) Effects of various metals (10 µg mL^−1^) on the detection of 0.3 µg mL^−1^ Pb(II) and Cd(II) in 0.1 acetate buffer with 2 M HNO_3_ and 5% HCl; (**B**) An effect of spiked (0.3 µg mL^−1^ of Cd(II) and Pb(II)) waste water from Dolni Rozinka deposit on detection of Cd(II) and Pb(II) in the same buffer as (**A**); (**C**) Dependence of metals extraction by 1 mL of 2 M HNO_3_ from 200 mg of soil in the time prolongation (1–10 min and 1–24 h). For all measurements *n* = 3.

In this section, we tested the effect of 10 µg mL^−1^ metals (Ni(II), Zn(II), Cu(II), As(III), Hg(I), Se(II), and Fe(II)) in buffer (0.1 M Acetate, 2 M HNO_3_, 5% HCl) on electrochemical detection of 0.3 µg·mL^−1^ of Pb(II) and Cd(II). It was found that these metals do not significantly affect the detection of Pb(II) and Cd(II), Figure 3A.

Further, we tested the effect of the matrix (waste water) on electrochemical response of Cd(II) and Pb(II). Detected values of Cd(II) and Pb(II) were below LOD and for this reason these are not shown. Buffer and three samples of water from Dolni Rozinka deposit were spiked by 0.3 µg·mL^−1^ of Cd(II) and Pb(II) solutions. It was found that the matrix does not have a significant impact on the electrochemical detection of Cd(II) and Pb(II), Figure 3B.

Finally, the effect of extraction time from soil sample was observed. Sample of soil (200 mg) was shaken in 1 mL of 2 M HNO_3_ (1–10 min and 1–24 h) and subsequently, the electrochemical responses of Cd(II) and Pb(II) were measured. It was proven that the concentration of Pb(II) increased with the increasing time of extraction (Figure 3C). The electrochemical response of Cd(II) cannot be recorded because the concentration of this metal was below LOD. In this manner, it was found that prolonged shaking time caused better extraction of Pb(II) for approximately 60% (10 h shaking), but already one-minute shaking may be sufficient for a tentative determination of lead in the soil, which may be subsequently subjected to further analysis.

### 3.4. On-Site Analysis of Soils Samples

**Figure 4:**
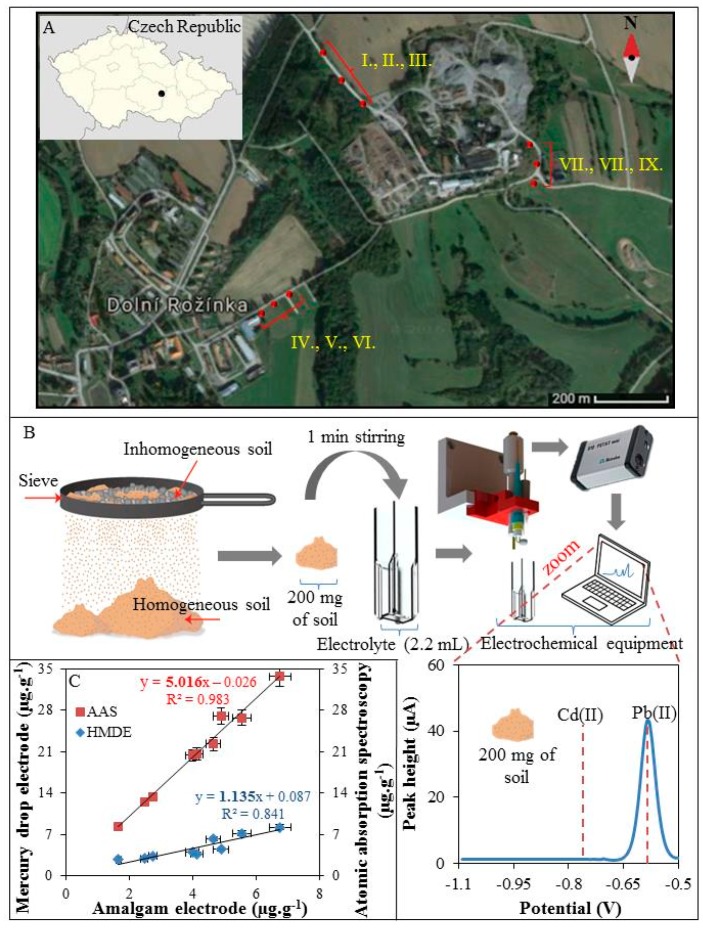
(**A**) Map of sample supply points (I–IX) along the transport roads of Dolni Rozinka; (**B**) Schematic representation of preparation and analysis of soil samples (step by step) under field conditions with real electrochemical record (voltammogram) of 200 mg of soil; (**C**) Correlation of two electrochemical methods used for the analysis of Pb(II) under field conditions (*x* axis) and in the laboratory (*y* axis) and AAS (*y* axis).

The samples were collected along the transport roads of Dolni Rozinka uranium deposit as surface samples of present soils and dust, (Figure 4A). Collected soils and dust (I–IX) were homogenized using a sieve with a mesh size of 2 mm. Then 200 mg of the soil sample was sprinkled into a plastic cuvette together with 1 mL of 2 M HNO_3_. The solution was thoroughly mixed (1 min) and then diluted by electrolyte (0.1 M acetate with 5% HCl (*v*/*v*)) to a total volume of 2.2 mL. Electrodes were immersed in to this suspension and Pb(II) and Cd(II) were detected (Figure 4B). Only Pb(II) was detected because the concentration of Cd(II) was below LOD. After completion of the analysis, the soils samples were transferred to the laboratory to verify the concentration of Pb(II) and Cd(II) by standard electrochemical method using a hanging mercury drop electrode and AAS. The individual concentrations of Pb(II) obtained from the proposed method using amalgam electrode and standard electrochemical method using mercury drop electrode and AAS were correlated. As it can be seen in Figure 4C, the concentrations of Pb(II) measured by electrochemical methods using amalgam electrode (Figure 4C, *x* axis) and the mercury drop electrode (Figure 4C, left *y* axis) are in good agreement. The standard method of elemental analysis (AAS) showed approximately five times higher sensitivity (Figure 4C, right *y* axis) compared to the method proposed here, however, it has to be highlighted that the AAS analysis has to be carried out in the laboratory using a relatively high cost and labour-demanding procedure. On the other hand, the complete decomposition of the sample by AAS and connected sample preparation process is able to extract metal ions also from the complexes with organic matter. This is impossible by the rapid and simple method presented here. Unfortunately, this is still a problem that needs to be addressed. Organic compounds interact with metal ions in various ways and prevent them from reduction at the electrodes during the process of electrochemical analysis [43]. Commonly, the samples are mineralized by microwave irradiation, assisted by acids [5], or irradiated by UV light [44]. For on-site applications, UV digestion is more applicable because it is easier to miniaturize and control. Also, the energy consumption of the UV LED or lamp is many times lower compared to the heating equipment. Baccaro et al. in his work presented an electrochemical cell containing a TiO_2_-modified gold electrode and the UV LED [45]. This device combines classical UV digestion with degradation of organics substances by radicals generated by TiO_2_ after UV irradiation and electrochemical detection. This technology is efficient and is therefore widely used in sewage treatment [46,47]. However, the applicability to different types of environmental samples and subsequently electrochemical detection has to be tested.

The typical mean Pb(II) concentration for surface soils worldwide averages 32 mg·kg^−1^ and ranges from 10 to 67 mg·kg^−1^ [48] and the content of Cd(II) in soil varies between 0.06 and 1.1 mg·kg^−1^ [49]. Our results suggest that the studied sites contain less Pb(II) than the world average. The average content of Pb(II) for all sites was determined to be 4 mg·kg^−1^, which is globally a very low concentration. In contrast, the average concentration of Cd(II) for sites was set at 0.05 mg·kg^−1^, which is under the global average value.

## 4. Conclusions

In this work, we showed a simple, rapid, and inexpensive method for preparation of an amalgam electrode, which can be used for a variety of voltammetry applications. This type of working electrode was used together with a portable potentiostat for detection of Pb(II) and Cd(II) in environmental samples near a uranium mine, located in the locality Dolni Rozinka (Czech Republic). This method can be used for analysis of the metals mainly in liquid samples, but because of easy extraction of metals with acids, it can be also applied for direct analysis of soil samples (complex matrix with an extremely low pH).

## Figures and Tables

**Table 1:** Analytical parameters of Cd(II) and Pb(II) determination.

Metal	Electrode Area (mm^2^)	Amalgamation Time (s)	Deposition Time (s)	Regression Equation	Linear Dynamic Range (ng·mL^−1^)	R^2^	LOD (ng·mL^−1^)	RSD (%)
Cd(II)	15	180	60	*y* = 95.302*x* − 6.9968	200–1143	0.996	80	8
Pb(II)	15	180	60	*y* = 111.64*x* − 5.0405	200–1143	0.993	50	7.5

LOD: Limit of Detection. RSD: Relative Standard Deviation of the signal intensity.
